# Biomarker dosimetry of acute low level of thermal neutrons and radiation adaptive response effect on rats

**DOI:** 10.1038/s41598-024-68640-z

**Published:** 2024-08-09

**Authors:** Misara M. Awad, Mahmoud H. Abdelgawad, Eslam Aboelezz, Khairy T. Ereiba

**Affiliations:** 1 Physics Department, Faculty of Science, Al-Azhar University, Cairo, Egypt; 2 Ionizing Radiation Metrology Department, National Institute of Standards (NIS), Giza, Egypt

**Keywords:** Thermal neutron, Adaptive response, Free radicals, Dosimetry, Antioxidant enzymes, Biomarker, Comet assay, Biophysics, Biomarkers, Energy science and technology, Physics

## Abstract

In this paper, we demonstrated the biological effects of acute low-dose neutrons on the whole body of rats and investigated the impact of that level of neutron dose to induce an in vivo radio-adaptive response. To understand the radio-adaptive response, the examined animals were exposed to acute neutron radiation doses of 5 and 10 mSv, followed by a 50 mSv challenge dose after 14 days. After irradiation, all groups receiving single and double doses were kept in cages for one day before sampling. The electron paramagnetic resonance (EPR) method was used to estimate the radiation-induced radicals in the blood, and some hematological parameters and lipid peroxidation (MDA) were determined. A comet assay was performed beside some of the antioxidant enzymes [catalase enzyme (CAT), superoxide dismutase (SOD), and glutathione (GSH)]. Seven groups of adult male rats were classified according to their dose of neutron exposure. Measurements of all studied markers are taken one week after harvesting, except for hematological markers, within 2 h. The results indicated lower production of antioxidant enzymes (CAT by 1.18–5.83%, SOD by 1.47–17.8%, and GSH by 11.3–82.1%). Additionally, there was an increase in red cell distribution width (RDW) (from 4.61 to 25.19%) and in comet assay parameters such as Tail Length, (from 6.16 to 10.81 µm), Tail Moment, (from 1.17 to 2.46 µm), and percentage of DNA in tail length (DNA%) (from 9.58 to 17.32%) in all groups exposed to acute doses of radiation ranging from 5 to 50 mSv, respectively. This emphasizes the ascending harmful effect with the increased acute thermal neutron doses. The values of the introduced factor of radio adaptive response for all markers under study reveal that the lower priming dose promotes a higher adaptation response and vice versa. Ultimately, the results indicate significant variations in DNA%, SOD enzyme levels, EPR intensity, total Hb concentration, and RDWs, suggesting their potential use as biomarkers for acute thermal neutron dosimetry. Further research is necessary to validate these measurements as biodosimetry for radiation exposure, including investigations involving the response impact of RAR with varied challenge doses and post-irradiation behavior.

## Introduction

Over the years, scientists have endeavored to monitor the effects of radiation at different levels and determine whether there exists a safe level of radiation. Radiation hormesis represents one of the observations of these studies. It is the concept that low doses of radiation might stimulate or positively affect living tissue^1^. According to the hypothesis of hormesis behavior, low doses of ionizing radiation, which is just above natural background levels, have a favorable effect, these doses stimulate the activation of repair mechanisms that protect against disease but remain inactive in the absence of ionizing radiation. Although the idea of radiation hormesis lost momentum with the change in known opinion, scientific analysis, and other external factors, it is worth mentioning that the linear non-threshold (LNT) model, built on high-dose exposure, was agreed upon for application to radiation protection and dose effects^2^. Even though the LNT model is generally accepted, there has been a resurgence of literature investigating the theory of radiation hormesis, containing valid epidemiologic studies on the important meaning of radiation hormesis in bacteria, plants, mammalian cells, and fungi. These studies have shown statistically significant differences for all various cell types investigated when exposed to low doses of radiation^3^. However, studies have shown that the low dose of ionizing radiation affects the repair of DNA. This impact ties the irradiation process to radiation adaptation, which speeds up the repair of DNA lesions and lowers the rate of new mutations in DNA^4^. Therefore, many scientific investigations in radiobiology and radiation biophysics aimed at understanding this phenomenon. Opposing results from both sides of the radiation hormesis argument indicate that the exact effects at low radiation doses are unknown^5^. According to a theory put forth^6^, the reserve repair mechanisms are sufficiently effective when activated to not only counteract the harmful effects of ionizing radiation but also to prevent disorders unrelated to radiation exposure. This concept has drawn interest from both the scientific community and the general public in recent years^7^. Among ionizing radiation types, neutrons can cause significant harm and toxicity to living cells and tissues.

However, the health risk of radiation exposure depends on the type of exposure. Acute exposure involves a substantial dose of radiation received within a short timeframe, resulting in immediate and delayed effects. Conversely, chronic exposure, which involves small, repetitive doses received over extended periods, may lead to delayed effects without immediate consequences^8,9^. Exposure to high doses of neutron radiation, exceeding 2 Gy, can result in acute radiation syndrome, radiation sickness, and even death^10^. However, research has demonstrated that exposure to low doses of neutron radiation can trigger an adaptive response in cells, enhancing their resistance to future exposure to higher doses of radiation^11^. Determining the impact of low neutron beams on the biological system is of particular importance, particularly in the realm of industrial safety for employees in nuclear reactors and oil-well logging. Accurately evaluating how neutrons affect living organisms is crucial due to the harmful effects of radiation on both humans and the environment, as well as the direct effects of neutron radiation. Thus, assessing the indirect effects of neutron radiation is also critical when evaluating risks^12,13^. Low-dose radiation biology has recently garnered more attention as a result of several studies indicating that Sister Chromatin Exchange (SCE) is predominant at doses below 100 mGy. The significance of doses below 50 mGy for radioprotection, as well as the numerous radiation-related applications for humans, including radiotherapy, has been addressed in favorable literature^14^. The signaling pathways that connect DNA damage to altered gene expression are complicated and can result in various gene expression profiles in response to numerous radiation exposure parameters. For example, gene expression forms have the potential to distinguish between fleeting low-dose-rate exposures and longer ones^15^.

When other physical dosimetry techniques are not available, biological dosimetry can be used to estimate the biological effects of ionizing radiation. Two fields of research in biological dosimetry deal with post-radiation incidents: final, rapid, high-throughput radiation dose assessment and triage-type radiation dose assessment^16^. Biomarkers used to assess biological doses must meet many requirements, including dose dependence, dose rate and radiation energy independence, and long-term stability. As all radiation dosimeters (physical, chemical, biological, or electronic) depend on the circumstances and specifications of the measurements, researchers are encouraged to discover new dosimeters and study their characterizations^17^.

The phenomenon of radiation adaptive response (RAR), also known as radio adaptation, may occur in an organism exposed to low doses of ionizing radiation. RAR is a fascinating subject of research in radiation biology, with potential implications for radiation protection and treatment of radiation-related diseases. El-Marakby et al.^18^ demonstrated that a radio-adaptive response occurs particularly after prolonged exposure to low-gamma doses before exposure to a relatively high dose. The RAR of low-dose neutron radiation has important implications for radiation protection and hazard assessment, as it suggests that exposure to low levels of radiation may be beneficial by inducing a protective response that reduces the risk of subsequent exposure. Additionally, the adaptive response may lead to new radiation protection strategies based on pre-exposure to low-dose radiation. Despite varied data on radiation risks, the health effects of acute low-level radiation exposure are still not fully understood. The impact study of low doses on normal cells and its potential benefits and drawbacks in medical applications is crucial. For instance, it can address the challenge of scattered radiation affecting the surrounding normal tissue when tumor cells require re-irradiation for recurrent tumor treatment using radiotherapy. In such cases, the accumulated radiation can expose the normal tissue to doses exceeding its limits, resulting in adverse effects. Therefore, the encouraging findings of the radio-adaptive response in this study warrant further exploration of how normal tissue surrounding tumor cells adapts to re-irradiation treatment.

In this study, we introduced two new terms, the Radio-Adaptive-Response Equivalent Dose (RARED) and Radio-Adaptive-Response Factor (RARF), to characterize the average changes in various biological parameters following exposure of mice to different high doses of irradiation after an acute priming dose. This factor can be used to quantify the changes in biomarkers resulting from a radio-adaptive response dose. Our study focuses on the effects of acute neutron radiation exposure at low levels of 5 and 10 mSv on some biophysical properties of rat blood. Is acute low-dose neutron exposure harmful? Do exposures to low priming dose of thermal neutrons before a high-dose thermal neutron exposure cause an RAR? Which parameters measured can be applied as biological dosimeters for acute doses of thermal neutrons?

## Materials and methods

### Animal preparation

Thirty-five adult male albino rats were selected for the experiments and were obtained from the Rat House at the National Research Center in Egypt. The chosen number of rats was committed according to the animal treatment ethics and resource availability. These animals have no significant difference in the mean weight which was 130 ± 10 g. They were housed a pathogen-free conditions cage with controlled temperature, 26 °C, and light (12-h day/night cycle) and free access to water and food. All animals were treated in accordance with the principles and guidelines of Laboratory Animal Facilities of the World Health Organization (WHO), Geneva, Switzerland^19^. Animals were randomly classified into seven groups, G1 is the control group (not irradiated), and the groups for G2–G7 exposure to different doses of neutron radiation will be explained in detail later. The present study was carried out in accordance with the Institutional Animal Care and Use Committee (IACUC), Egypt, and the treatment of animals was approved by the Institutional Animal Care and Use Committee.

### Description of irradiation setup

**Figure 1:**
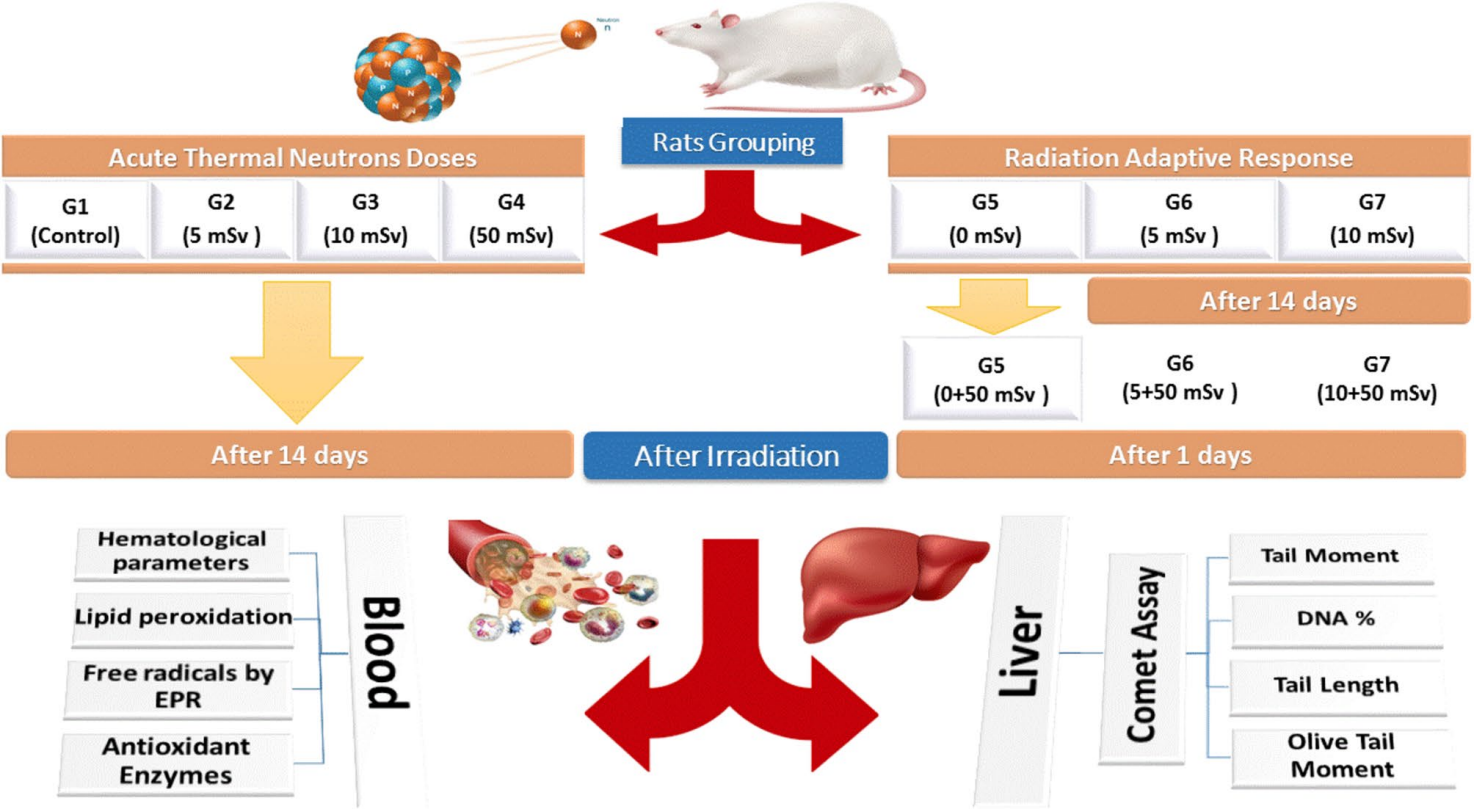
Scheme of thermal neutrons irradiation and different planned parameters for measurements. The exposure was from an Am–Be source with an equivalent dose rate of 1.5 ± 0.06 mSv/h for all classified rat groups.

The irradiating system consists of five components: neutron source (Am–Be, 5Ci), neutron moderator (polyurethane, ARTELON®), gamma shield (lead), rat cage and source handling system. Fast neutrons were moderated by ARTELON delivering thermal neutron doses from the Am-Be source to rats as shown in Fig. 1. The neutron source emits spectrum neutrons from 0 up to 11 MeV with an average energy of around 4.5 MeV. The neutron dose equivalents are measured using a secondary standard neutron monitor (Nuclear Enterprise, UK, NM2) at the National Institute of Standards (NIS), Giza, Egypt, that is traceable to the SI unit and calibrated at PTB, Germany^20^. All rats were irradiated to neutron with a dose rate of 1.5 mSv/h. Nearly all gamma rays emitting from the neutron source were eliminated using lead sheets, with a dose rate of 11 mSv/h for g-rays which is a good shielding material for gamma rays and other electromagnetic radiation, whereas lead is a poor shielding for thermal neutrons. The thickness of the used lead in the shielding was about $8.5\pm 0.2$ cm. This study focused on exposure rats to low doses of acute neutron radiation exposure, time, duration, and distance from the source under good ventilation with regular feeding and drinking water.

### Animal exposure to acute neutron radiation

After categorizing the animals into seven groups, Group #1 served as the sham control group. This group involved placing the rats in the same cages as those exposed to neutrons for same duration but without irradiation. Rats in groups #2 and #6 were exposed to acute neutron radiation with a total dose of 5 mSv of neutron radiation. After 14 days, group #6 was exposed to a single challenge dose of 50 mSv. For group #4, rats were exposed to only 50 mSv without exposure to neutron radiation. Then, 14 days later, rats were harvested for examination. In groups #3 and #7, rats were exposed to 10 mSv acute neutron radiation, while group #7 was exposed to a single challenge dose of 50 mSv. Whereas in group #5, rats were exposed to only 50 mSv, which was marked as a control group for RAR. After 24 h of the irradiation, rats were harvested for examination. In addition, the exposure of each group did not affect the other groups. Table 1 summarizes the plan for animal irradiation.

**Table 1:** Irradiation plan to different thermal neutron doses for all classified groups.

Group	Neutron dose*	Period between two irradiations (days)	Challenge dose (mSv)	Time before measurements (hr)
G1 (Acute control)	Non	Non	Non	24
G2	5	14	Non	24
G3	10	14	Non	24
G4	50	14	Non	24
G5 (RAR control)	Non	Non	50	24
G6	5	14	50	24
G7	10	14	50	24

*The equivalent dose rate of thermal neutrons is 1.5 mSv/h.

### Hematological analysis

Blood samples were collected for hematological analysis in tubes containing EDTA (Anti Coagulate) by eye puncture via a capillary tube and were analyzed within two hours of collection^21^. The analysis of hemoglobin concentration (Hb), mean corpuscular volume (MCV), hematocrit (HCT), Red Cell Distribution Width (RDW), and mean corpuscular hemoglobin concentration (MCHC), a hematology Analyzer (Diff3) Mek6410/Mek-6420 was used, following Wintrobe's technique^22^.

### Lipid peroxidation analysis

The blood serum was used for lipid peroxidation analysis. In this test, blood samples were collected in plain tubes without anti-coagulant. The samples were then centrifuged for 10 min at 3000 rpm to separate the serum which was stored in a refrigerator at—20 °C for one week until used. To measure the lipid peroxidation product, malondialdehyde (MDA), a Thiobarbituric acid assay was performed. This assay is based on MDA's reaction with Thiobarbituric acid, resulting in Thiobarbituric acid reactive substances (TBARS), a red species that absorbs at 532 nm^23^.

### Electron paramagnetic resonance (EPR) analysis

EPR measurements were performed using an X-band EPR spectrometer (Bruker EMX, Germany) at room temperature using a high-sensitive standard cylindrical resonator (ER4119HS) operating at 9.85 GHz, with a 100 kHz modulation frequency. The optimum EPR parameters were 2 mW microwave power, the modulation amplitude was 1 G, and the response time constant was 20 ms with a sweep time was 84 s. The optimum weight of dried blood powder was around 40 mg to fit 10 mm in height inside the tube. The EPR intensity, peak-to-peak height, of each lyophilized blood sample at room temperature by freezing and drying the resulting solution, was measured 3 times with 5 scans each run. The average values of the obtained measurements were normalized to weight and then plotted. Before all measurements, the standard sample of MgO doped with Mn^2+^ was utilized independently to calibrate the EPR intensity, spectrometer stability, and the signal g-factor.

### Biochemical examination

Similar to lipid peroxidation analysis, the serum was isolated in separate tubes without anti-coagulant materials for the biochemistry examination. It was performed by centrifuging the sera for 10 min at 3000 rpm and then storing it in a refrigerator at − 20 °C for almost one week before measurements. Catalase activity (CAT) was determined using Sinha 1972 approach by assessing the catalytic reduction of hydrogen peroxide^24^. The superoxide dismutase (SOD) activity was determined using Minami and Yoshikawa 1972 technique^25^. While Ahmed et al.^26^ employed the method to measure the reduced glutathione (GSH) content.

### Comet assay analysis

To detect the alkaline comet or single-cell gel electrophoresis (SCGE) assay, the technique reported by Singh et al.^27^ was employed with slight changes to the measurement of comet assay (CA), tail length (TL), tail moment (TM), percentage of DNA in the tail (DNA%), and olive tail moment (OTMS). Due to the liver being an active organ in various biochemical processes in addition to the liver cells being sensitive to the DNA damage induced by radiation, the liver cells were chosen for the comet assay analysis. Following the animals sacrificing, 20 g of liver organs were collected and mixed with 10 ml of cold phosphate buffer solution (PBS) and centrifuged again for 15 min at 4000 rpm at 4 °C. The tissues were dried with sterilized absorbent paper. To prevent DNA damage, the liver cells were added to low-melting agarose. Approximately 1 × 10^4^ cells were combined with low melting agarose (Solarbio, Beijing, China) and dispersed on microscope slides. The cells were lysed at pH 10, then the DNA was unwound for 20 min in an alkaline buffer (pH 13), followed by 30 min of electrophoresis (25 V, 300 mA). After neutralization (5 mg/L, Sigma, St. Louis, MO), the cells were stained with ethidium bromide. We utilized a fluorescence microscope (Nikon, Tokyo, Japan) loaded with CASP1.2.3 beta 1 software, developed by CaspLab-Comet Assay Software Project Lab which is freely available at: (https://sourceforge.net/projects/casp/) to assess all coded slides for each exposure condition. We measured the TL of 50 comets in micrometers, while TM was calculated from the comet tail length multiplied by the DNA percentage in the comet tail^28^. The data was decoded after completing all microscopic analyses.

### RAR equivalent dose and RAR factor calculation

The term "RAR equivalent dose, RARED, (D_RAR_)” introduces a new concept indicating the actual equivalent dose with respect to changes in the investigated biological parameters due to the response of radio-adaptation. This definition allows for the measurement of animal ability to resist the effects of a larger challenge dose. It does so by comparing the extent of change in certain parameters when exposed to a high challenge dose after receiving small, acute priming doses with the change in these parameters after exposure to small, acute doses only. This ability can be measured quantitively by a new factor called “RAR factor, RARF, (${f}_{RAR}$)”, which is calculated using the following equation:

1 $$f_{RAR} = 1 - \left( {(D_{RAR} - D_{p} )/D_{p + c} } \right)$$

where (${D}_{p}$) is the priming acute dose only and (${D}_{p+c}$) is the priming and challenge dose.

Equation (1) is derived in a similar manner to the supposed equation of Yonezawa effect scheme^29^, which is known as “the priming dose model”, used for calculating the difference percentage due to radio-adaptation (δ) as in Eq. (2)^30^.

2 $$\delta = 1 - \left( {N_{p + c} /N_{c} } \right)$$

where ${(N}_{p+c})$ is the reading of the marker irradiated to priming and challenge doses and $({N}_{c})$ is the reading of the challenge dose without the priming dose. At constant intervals between the priming and challenge doses and also between the challenge dose and the readout of markers, we proposed here an equation as a function of the equivalent doses only. RARED and RARF of acute doses of thermal neutrons to the radio-adaptive response doses proposed here were calculated from the calibration curve by fitting the measured marker data points. The RAR factor (${f}_{RAR}$) values range from 0 to 1. A value of zero indicates no RAR effect, while a value of 1 represents a full radio adaptive response (i.e. there is no scientific variation in the biomarkers between the zero dose and priming acute dose). This also means that the effect of RAR increases as the RARF increases from 0 to 1.

### Statistical analysis

During our statistical analysis, we calculated the average results of five rats in each group, in addition to standard deviation (SD) values. Furthermore, we determined the degree of significance for *p* ≤ 0.05, where a difference is considered significant and meets the tolerance criteria. The values in the goodness of fitting column shown in all tables summarizing the fitting parameters of investigated biomarkers refer to *p*-values. Our approach involved the use of the statistical OriginPro 2021b program, developed by OriginLab (https://www.originlab.com/), to fit all data. This included calibration functions and dose–response relationships for all parameters of acute neutron doses to radiation (5, 10, 50 mSv), and their reverse functions.

### Declaration of animal treatment ethics

The authors declare that the animals were treated based on the guidelines of the National Institute of Health for human treatment of animals (USA) and the institutional animal care and use committee approved the work under approval number URAF-2-23.

## Results

### Antioxidant enzymes for the blood

**Figure 2:**
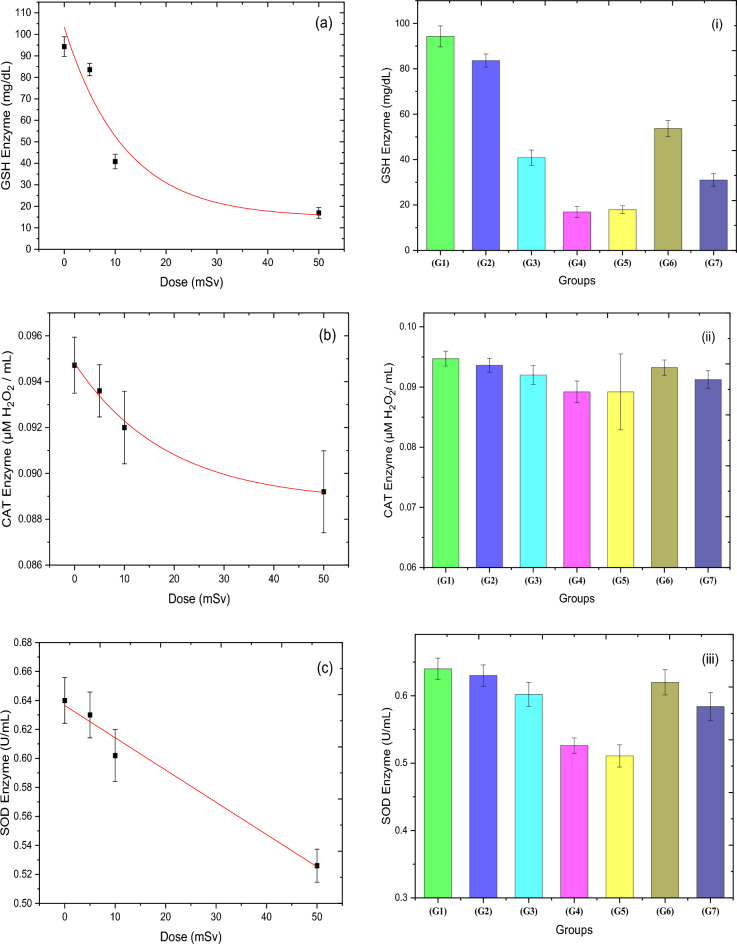
Shows the effect on the (**a**) antioxidant enzymes [GSH], (**b**) [CAT], and (**c**) [SOD] for the four groups (G1, G2, G3, and G4) that were exposed to different acute doses of thermal neutrons (0, 5, 10 and 50 mSv), respectively. (i)–(iii) demonstrates overall effects for all groups on antioxidant enzymes [GSH], [CAT], and [SOD], respectively.

Figure 2 describes the effect of thermal neutrons on three antioxidant parameters: CAT, SOD, and GSH. As appeared in this figure, the exponential decay fitting of both GSH and CAT concentration for the groups that irradiated to 5, 10, and 50 mSv (G4) acute thermal neutron doses has a significant decrease in comparison to G1, ranging from 11.3 to 82.1% and from 1.18 to 5.83%, respectively, for G2 and G4. Also, there was a significant linear decrease (see Table 2) in the concentration of SOD enzyme for acute neutron doses when compared to the control group, as follows: 1.47, 6.09, and 17.8%. For RAR, a significant increase in all concentrations of antioxidant enzymes was observed for the 5 + 50 mSv groups (G6), where the values of GSH, CAT, and SOD increased by 200, 4.51, and 21.38%, respectively, in comparison to G5. While for G7, they have a slight and significant increase relative to G5, with a percentage of 73, 2.29, and 14.33%, respectively. Amongst the enzymes under investigation, the GSH enzyme has the highest response due to exposure to thermal neutron doses; moreover, it shows the best indication for RAR.

**Table 2:** The fitting parameters of the relation between antioxidant enzymes and neutron dose for the groups that were exposed to an acute dose of neutron radiation in addition to the control (zero dose) group.

Enzymes	Type of fitting	Equation	Equation constants	R-value	Goodness of fitting
GSH	Exponential	$y=A{e}^{({R}_{0} x)}+{y}_{0}$	${y}_{0}=14.8\pm 15.78$ $A=88.43\pm 26.17$ ${R}_{0}=-0.085\pm 0.062$	$0.962$	Fit converged*
CAT	Exponential	$y=A{e}^{({R}_{0} x)}+{y}_{0}$	${y}_{0}=\left(88.8\pm 0.77\right){\times 10}^{-3}$ $A=\left(6.05\pm 0.75\right){\times 10}^{-3}$ ${R}_{0}=-0.054\pm 0.018$	$0.995$	Fit converged*
SOD	Linear	$y=ax+b$	$a=\left(-22\pm 1.78\right){\times 10}^{-4}$ $b=0.6365\pm 0.00576$	$-0.994$	0.006

*Tolerance criterion satisfied.

### Lipid peroxidation in the blood

**Figure 3:**
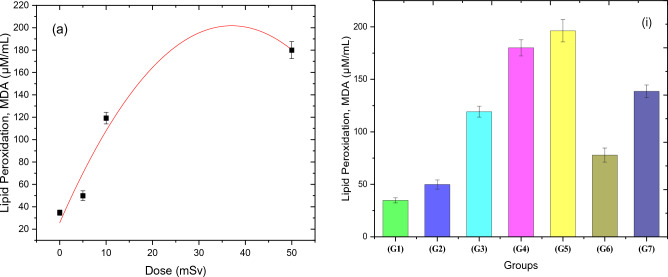
(**a**) Shows the effect of acute neutron doses (0, 5, 10, and 50 mSv) on MDA concentration for the four groups (G1,2,3,4). The polynomial fit of relationship between the lipid peroxidation (MDA) and the acute neutron radiation dose was performed. (i) shows overall behavior of lipid peroxidation process for all groups. Error bars represent the standard deviation of five aliquots.

Figure 3a explains the effect of lipid peroxidation (MDA) on G1, G2, G3, and G4 as a result of exposure to acute doses of thermal neutron, while Fig. 3(i) depicts the effects of lipid peroxidation on all groups under investigation. As indicated in Fig. 3a, when compared to the control group, there is an elevation in the rate of the peroxidation process, but with a non-significant polynomial increase. Polynomial increase of lipid peroxidation in Fig. 3a for 5, 10, and 50 mSv (G4) neutron doses, with R = 0.9331, and the fitting parameters of this relation were summarized in Table 3. The MDA intensity of the G6 and G7 groups is less than that of G5, which was irradiated to a 50 mSv challenge dose (RAR), by a value of 60.34% and 29.38%, respectively.

**Table 3:** The fitting parameters of the relation between dose and Lipid Peroxidation for the groups that were exposed to acute doses of neutron radiation in addition to the control (zero doses) group.

Parameter	Type of fitting	Equation	Equation constants	R-value	Goodness of fitting
MDA	Polynomial	$y={intercept+B}_{1}{X}_{1}+{B}_{2}{X}_{2}$	$intercept=31.93\pm 13.49$ ${B}_{1}=8.15\pm 3.7$ ${B}_{2}=-0.103\pm 0.0741$	$0.9331$	$0.258$

### Free radical intensity in the blood

**Figure 4:**
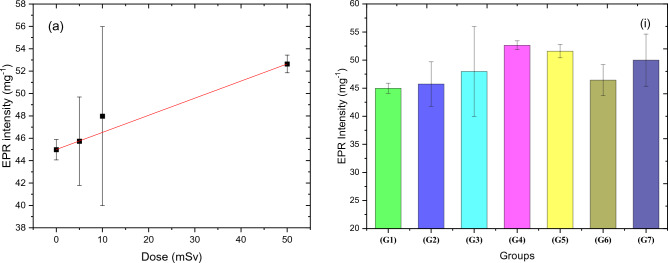
(**a**) The relation between EPR intensity for the four groups (G1, G2, G3, and G4) and the acute neutron equivalent dose. (i) shows overall changes on EPR intensity for all groups, indicating the adaptation response.

The relationship between the concentration of radiation-induced free radicals, measured as EPR intensity, in the blood and acute doses of thermal neutrons, as well as the impact of the neutron dose on all groups, including the RAR effect, are portrayed in Figs. 4a and i, respectively. EPR intensity for 5, 10, and 50 mSv (G4) neutron doses increased linearly, with R = 0.9996, and the fitting parameters of this relation were summarized in Table 4. The EPR intensity of the G6 and G7 groups is less than that of G5, which was irradiated to a 50 mSv challenge dose, by a value of 10% and 3.1%, respectively. This indicates obviously that the adaptation response after exposure to 50 mSv of thermal neutron occurred.

**Table 4:** The fitting parameters of the relation between EPR intensity and dose for the groups that were exposed to an acute dose of neutron radiation in addition to the control (zero dose) group.

Parameter	Type of fitting	Equation	Equation constants	R-value	Goodness of fitting
EPR	Linear	$y=ax+b$	$a=0.153\pm 0.003$ $b=45\pm 0.114$	$0.9996$	$4.03\times {10}^{-4}$

### Double and single strand DNA on rats using single cell gel electrophoresis (comet assay)

**Figure 5:**
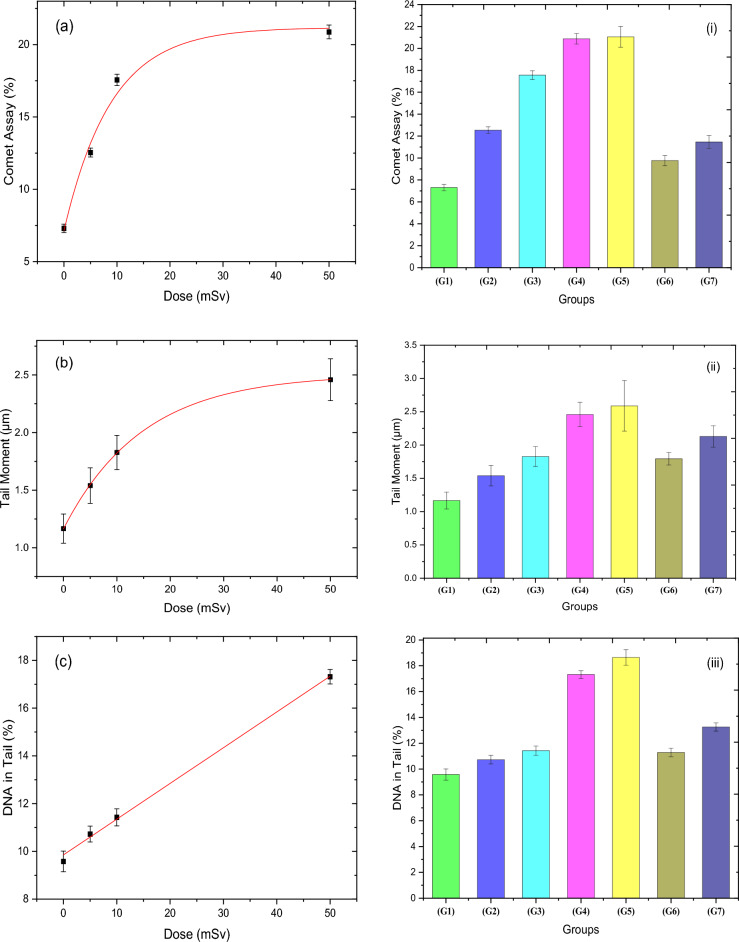

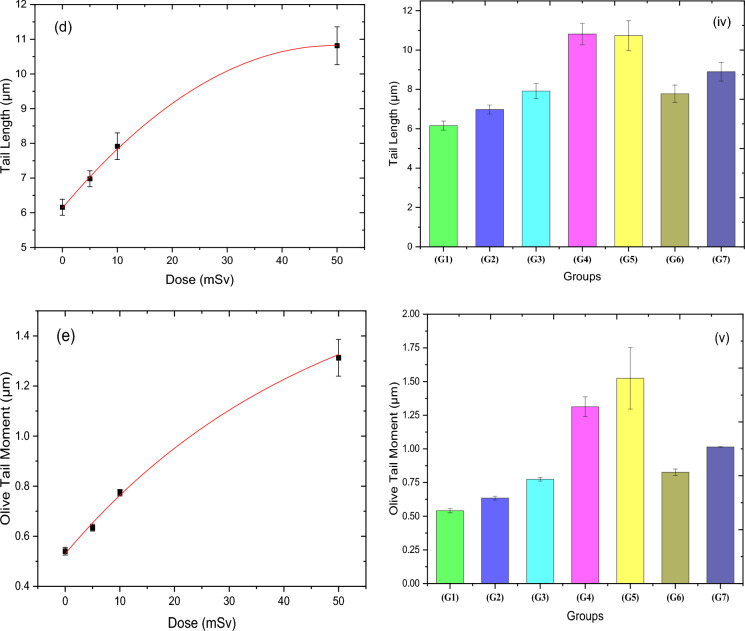
(**a**–**e**) show the comet assay parameters for liver cells [CA], [TM], [DNA%], [TL] and [OTM], respectively, for the four acute dose groups (G1, G2, G3, and G4) that were exposed to acute neutron radiation for doses (0, 5, 10, 50 mSv). The impact of neutron dose on all groups as demonstrated in (i–v) for [CA], [TM], [DNA%], [TL], and [OTM], respectively.

Figure 5 demonstrates the dose–response of comet assay percentage (CA%), tail moment (TM), percentage of DNA in the tail (DNA%), tail length (TL), and olive tail moment (OTM) for the irradiated groups. Table 5 shows the fitting parameters of the acute neutron dose response for each measured factor of the Comet Assay technique. As it is clear from this figure, all Comet assay parameters for liver cells revealed an increase in the exposed groups to neutron doses of 5 mSv (G2), 10 mSv (G3), and 50 mSv (G4). The dose–response behavior for the CA% and TM factors is both fitted exponentially, increasing from 7.3 to 20.87% and from 1.17 to 2.46 µm, respectively. While DNA% rises significantly in a linear response as the neutron dose increases, from 9.58 to 17.32%. On the other hand, polynomial fitting for both TL and OTM was the best choice of the fitting option; TL increased from 6.16 to 10.81 µm, and OTM increased from 0.54 to 1.31 µm. Regarding the RAR study, all comet assay factors showed an increase for the 5 + 50 mSv (G6) and 10 + 50 mSv (G7) groups by a value of as follows: CA% (9,76, 11,46) %, TM (1.79, 2.13) µm, %DNA (11.28, 13.26) %, TL (7.87, 8.9) µm, and OTM (0.83, 1.014) µm, respectively. However, by comparing the same two previous groups to the group that was exposed to 50 mSv (G5) only, their values are decreased for all comet assay parameters by the following percentages: CA% (53.6, 45.5)%, TM (30.7, 17.8)%, DNA% (39.5, 28.9)%, TL (27.5, 17.1)%, OTM (45.8, 33.5)%, indicating the RAR behavior of the rat against the low doses of the thermal neutron.

**Table 5:** the fitting parameters of the dose response of comet assay parameters (CA%, TL, DNA%, TM, and OTM) for the acute dose groups in addition to control (zero dose) group.

Parameter	Type of fitting	Equation	Equation constants	R-value	Goodness of fitting
CA%	Exponential	$y=A{e}^{({R}_{0} \times X)}+{y}_{0}$	${y}_{0}=21.18\pm 1.589$ ${R}_{0}=-0.112\pm 0.0297$ $A=-14.04\pm 1.803$	$0.9996$	Fit converged
TM	Exponential	$y=A{e}^{({R}_{0} \times X)}+{y}_{0}$	${y}_{0}=2.506\pm 0.015$ ${R}_{0}=-0.067\pm 0.002$ $A=-1.342\pm 0.016$	$0.9999$	Fit converged
DNA%	Linear	$y=ax+b$	$a=(149\pm 4.5){\times 10}^{-3}$ $b=9.852\pm 0.131$	$0.991$	$8.9\times {10}^{-4}$
TL	Polynomial	$y={intercept+B}_{1}X+{B}_{2}{X}^{2}$	$intercept=6.136\pm 0.0724$ ${B}_{1}=0.1886\pm 0.0167$ ${B}_{2}=(-19\pm 3.26){\times 10}^{-4}$	$0.9992$	$0.0402$
OTM	Exponential	$y=A{e}^{({R}_{0} \times X)}+{y}_{0}$	${y}_{0}=1.741\pm 0.564$ ${R}_{0}=-0.0214\pm 0.0128$ $A=-1.211\pm 0.556$	$0.993$	Fit converged

### Blood hemoglobin and some blood parameters

**Figure 6:**
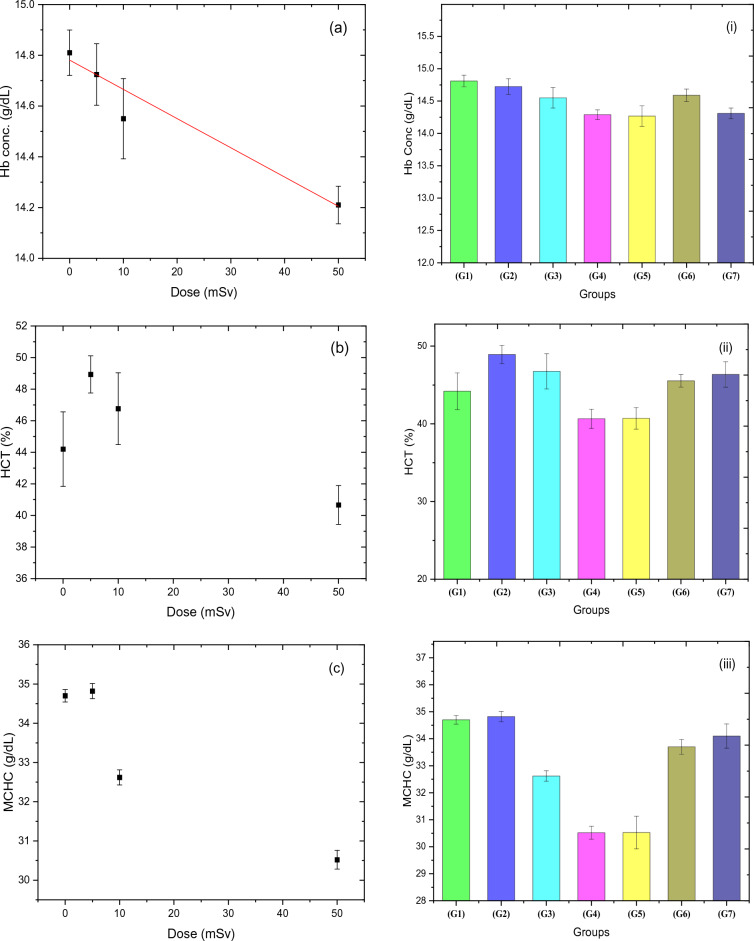

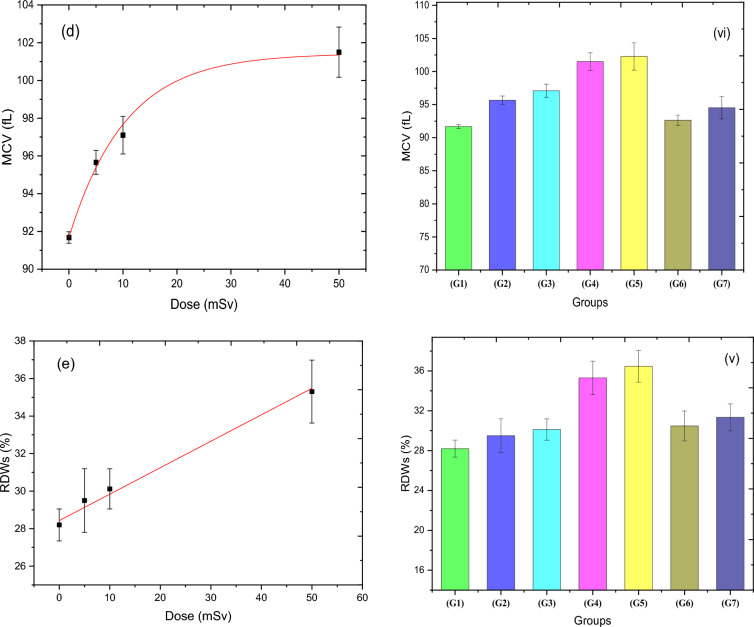
(**a**–**e**) illustrate the effects of acute neutron radiation at doses of 0, 5, 10, and 50 mSv on the Hb parameters (Hb conc.), (HCT), (MCHC), (MCV), and (RDWs), respectively, for the four groups (G1, 2, and 3), demonstrating the direct correlation between the Hb parameters and the acute neutron radiation dose. (i–v) show the RAR behavior the responses of Hb parameters to thermal neutron (Hb conc.), (HCT), (MCHC), (MCV), and (RDWs), respectively, for all groups.

The effect of various acute doses of thermal neutron on the concentration of some blood parameters (total Hb, HCT, MCHC, MCV, and RDWs) is presented in Fig. 6a–e for groups 1, 2, 3, and 4. Both Hb and RDWs, as shown in Fig. 6a and e have a significant change and were fitted linearly, where the slope of the regression line for Hb has a negative sign in contrast to that of RDWs, which has a positive value (see Table 6). The percentage of change in Hb concentration, relative to the control group, fell gradually till reached about 3.5% for 50 mSv, while for RDWs, it increased dramatically to 25.2% for the same dose. Whereas the MCV factor (Fig. 6d) has an exponential trend as a response to the neutron dose. On the other hand, HCT and MCHC indices have a fluctuated response, as noticed in Fig. 6b and c. Figure 6i–v depicts the responses of all groups, including the RAR groups study, demonstrating that the responses of both 5 + 50 mSv (G6) and 10 + 50 mSv (G7) groups, in all parameters except HCT and MCHC, have not followed the same trend of dose–response as the acute dose groups (G1–G4). The percentages of concentration change for MCV, and RDWs in G6 and G7 groups have a lower value when compared to G5 by (9.4 and 7.6)% and (16.4 and 14)%, respectively. Contrarily, total Hb has a higher concentration to some extent by a value of 2.3% for G6 and 0.3% for G7. This unambiguously indicates that all blood parameters under study, except HCT and MCHC, confirm the adaptation response behavior of rats exposed to 5 or 10 mSv prior to a relatively high dose of thermal neutrons.

**Table 6:** the fitting parameters of the relation between dose and Hb parameters (Hb%, MCV, RDWs) for the groups that were exposed to an acute neutron radiation in addition to the control (zero dose) group.

Parameter	Type of fitting	Equation	Equation constants	R-value	Goodness of fitting
Hb conc	Linear	$y=ax+b$	$a=(-115\pm 12){\times 10}^{-4}$ $b=14.78\pm 0.039$	$-0.979$	0.011
MCV	Exponential	$y=A{e}^{({R}_{0} \times X)}+{y}_{0}$	${y}_{0}=101.4\pm 1.035$ $A=-9.73\pm 1.05$ ${R}_{0}=-0.095\pm 0.02$	0.997	Fit converged
RDWs	Linear	$y=ax+b$	$a=(141\pm 11.7){\times 10}^{-3}$ $b=28.43\pm 0.215$	$0.9931$	$0.007$

### RAR equivalent dose (RARED) and RAR factor (RARF)

The values of RARED were estimated from the calibration curve used for the fitting of measured marker data points and as a consequence, RARF values are calculated using Eq. (1) and summarized in Table 7. The mean values of these RAR equivalent dose for 5 + 50 mSv vary from 5.6 to 16.6 mSv for CAT and Hb parameters, respectively, with an average of $10.1 \pm 3.4$ mSv ($\sigma =1$), while the values for 10 + 50 mSv range from $16.5$ to $40.9$ mSv for the same parameters, respectively, with an average of $23.1 \pm 7.5$ mSv. The percentages of the standard variation between readings of G4 and G5 for all parameters studied in this paper are less than $4\%$, except OTM, DNA%, and MDA, which reach approximately $10\%$. On the other hand, the RARF values, as seen in Table 7, range from 0.79 for Hb to 0.99 for CAT, with an average of 0.91 ± 0.06 for 5 + 50 mSv. For the 10 + 50 mSv range, the values range from 0.49 for Hb to 0.89 for CAT, with an average of 0.78 ± 0.12. These results provide valuable insights for further analysis and interpretation.

**Table 7:** The mean of the equivalent dose of RAR and the calculated factor of RAR for each marker under the study as an acute neutron dose for 5 + 50 and 10 + 50 mSv doses.

Mean of RARED (mSv) and RARF														
Dose	Parameters	GSH	CAT	SOD	MDA	EPR	TM	DNA%	TL	OTM	Hb	RDWs	Average	SD
(5 + 50 mSv)	RARED	9.7	5.6	7.4	6.11	9.4	9.4	9.6	9.6	13.1	16.6	14.6	**10.1**	**3.4**
	RARF	0.91	0.99	0.96	0.98	0.92	0.92	0.92	0.92	0.85	0.79	0.83	**0.91**	**0.06**
(10 + 50 mSv)	RARED	19.9	16.5	23.6	16.6	32.6	18.8	22.8	17.9	23.8	40.9	20.6	**23.1**	**7.5**
	RARF	0.84	0.89	0.77	0.89	0.62	0.85	0.79	0.87	0.77	0.49	0.82	**0.78**	**0.12**

Average and standard deviation values are in bold.

## Discussion

In the realm of radiation exposure, antioxidant enzymes are vital in protecting cells from the harmful effects of free radicals^31^, Reactive oxygen species (ROS) are removed by these enzymes to safeguard organisms from oxidative damage^32^. ROS is important for several biological processes, including cell differentiation, growth regulation, immunity, and programmed cell death^33,34^, and defense against microorganisms^35^. Ionizing radiation generates various types of ROS, such as superoxide, hydrogen peroxide, and hydroxyl radicals, which can cause severe damage to proteins, lipids, and nucleic acids. Furthermore, radiation damage to lipids is more severe due to the lipid component in the membrane^36^. The body's defense mechanisms against oxidative stress caused by free radicals include preventive defenses, repair defenses, physical defenses, and antioxidant defenses. Enzymatic antioxidants, such as glutathione peroxidase (GSH), catalase, and superoxide dismutase (SOD), form a critical component of the antioxidant defense system. Under normal conditions, a balance between the intracellular levels and the activity of these antioxidants is maintained. The accumulation of free radicals due to an imbalance between antioxidants and oxidants can damage macromolecules, leading to abnormal gene expression and various disease conditions.

The survival of organisms and their health depend on this equilibrium^37^. Antioxidants become oxidized and need to be replaced or renewed. Antioxidant enzymes, often found in cells, catalyze the destruction of several free radical species. Transition metal binding proteins inhibit the formation of extremely reactive hydroxyl radicals by blocking the interaction of transition metals such as iron and copper with hydrogen peroxide and superoxide, converting them into new products. Therefore, the decline in the concentrations of antioxidant enzymes can be attributed to their conversion into new products. As the equivalent dose of neutron increases, so do the concentrations of free radicals, which require more antioxidants to deactivate these radicals, resulting in a further drop in the concentration of antioxidants in the blood. There are numerous antioxidants, including GSH, CAT, SOD, and others^38^, which are effective scavengers of free radicals by giving electrons to ROS. They neutralize the negative effects of the latter, reducing oxidative stress and the oxidation of cell molecules^39^. The lack of antioxidant enzymes causes excessive oxidative stress, which increases the risk of disorder and negative treatment outcomes^40^. In response to an increase in free radicals, cells produce extra endogenous antioxidants such as catalase, glutathione, and superoxide dismutase that can reduce or eliminate damage to cell structure. SOD reduces superoxide ions to hydrogen peroxide, which is converted to water by catalase, while glutathione peroxidase lowers hydroxide ions^41^.

The effects of whole-body sub-lethal doses of gamma-ray exposure on plasma lipids and susceptibility to oxidative stress were studied in rats^42^. A significant elevation in MDA level and a reduced level in GSH content were found after radiation exposure indicating disorders of lipid metabolism. We studied the enzyme activities to confirm their contributions to the adaptive response. The results of the present study showed a low production of antioxidant enzymes that can be fitted exponentially in GSH and linearly in CAT and SOD in acute neutron exposure for both 5 and 10 mSv, then deep decreasing in the group of exposure to 50 mSv (G4) only. The exposure to 5 + 50 mSv (G6) of neutrons resulted in elevated concentrations of the antioxidant enzymes GSH, CAT, and SOD, accompanied by a decrease in free radicals when compared to 50 mSv (G5), which represented an adaptive response in rat blood cells. The RARED were 9.7, 5.6, and 7.4 mSv and RARF were 0.91, 0.99, 0.96 for GSH, CAT, and SOD, respectively.

Lipid peroxidation is a natural and necessary process that occurs in all cells and tissues at low levels, producing MDA and other byproducts^43^. Measuring MDA levels helps determine the impact of ROS on biological systems. Lipid peroxidation, which is caused by free radicals, is a process that oxidizes polyunsaturated fatty acids. Elevated lipid peroxides can interface with the biochemical and physiological processes of red blood cells^44^. Researchers investigated the radioprotective effect of recombinant human antioxidant enzymes on mice subjected to 5–7 Gy γ-irradiation. The study showed that rhCuZn-SOD had a significant radioprotective effect by eliminating free radicals, increasing the activities of SOD, GSH, and CAT in blood and liver cells, reducing MDA levels, enhancing the immune system response, and prolonging survival. Excess free radicals can lead to cell death, and exposure to gamma radiation can increase MDA levels^45^. The deleterious effects of excess free radicals, or oxidative stress, have been reported to eventually lead to cell death. Benderitter et al.^46^ noted an increase in malondialdehyde MDA a few hours after gamma radiation exposure. Lipid peroxidation results showed an increase as the acute neutron dose increased, which is expressed using a quadratic polynomial function. Also, it showed a deep decrease in the lipid peroxidation process in the group of 5 + 50 mSv and a slight decrease in the 10 + 50 mSv group when compared to a challenge dose of 50 mSv (G5) only, indicating the occurrence of an adaptive response.

EPR is the only non-destructive method that directly detects paramagnetic species, such as free radicals^47^. This makes it an effective tool for determining the concentration of unpaired electrons in a sample, even if the specific free radical is unknown. Additionally, EPR can identify biological molecules that contain free radicals or transition metal ions (Fe^3+^, Cu^2+^, Mn^2+^, and Co^2+^)^48^. Neutron, gamma, or X-ray radiation exposure stimulates the body to create free radicals. The results of our study indicate a slight increase in the production of free radicals in all acute exposure groups (G2, G3, and G4) for doses (5, 10, and 50 mSv). Although exposure to a neutron dose of 5 mSv caused higher free radicals, it showed a higher radio adaptive response through lower free radical density at exposure to 50 mSv after 5 mSv compared to exposure to a neutron radiation dose of 10 + 50 mSv.

The comet assay (CA) is a versatile, uncomplicated, and adaptable technique for evaluating DNA damage and repair at the cellular level. The CA enables the detection of early or acute DNA damage after brief exposure, which may be repaired or subjected to programmed cell death (apoptosis) and/or mutations, leading to less detectable DNA damage^49^. Radiation-related cell damage is also known to involve the formation of free radicals, including hydroxyl radicals, lipid peroxide radicals, superoxide radicals, and lipid radicals. Lipid peroxide radicals cause lipid peroxidation in biological membranes, resulting in various biological damages, along with direct DNA damage^50^. The hydroxyl radical reacts with all components of the DNA molecule, causing damage to both the purine and pyrimidine bases and the deoxyribose backbone^51^. Kumaravel and Jha^52^ used a comet assay to detect the measure(s) most strongly linked to DNA damage following exposure to various levels of gamma radiation ranging from 1 to 8 Gy. The study recorded various parameters, including OTM, TL, TM, DNA%, and others. Despite the increasing trend of these parameters, the results in that study are not comparable to those of our current study because of the variation in the range of gamma dose. The comet test revealed less DNA damage in the cells of mice exposed to pre-irradiated gamma rays than in the cells of rats that only received the challenge dose^53,54^.

Adaptive Response is becoming increasingly important in biodosimetry or accidental risk assessment for occupational workers and radiotherapeutic patients, which are assayed on CA mutations as endpoints. The present study showed an increase in all comet assay parameters (TM, DNA%, TL, and OTM) after exposure to acute doses of neutron radiation. Low-dose irradiated groups (G2 and G3) showed lower DNA damage when compared to the irradiated groups with a challenge dose of 50 mSv (G6 and G7). The adaptive response was demonstrated upon exposure to neutron radiation for 5 mSv before the challenge dose of 50 mSv, yet it is less obvious after neutron exposure for 10 mSv before the 50 mSv challenge dose. The adaptive response was undoubtedly determined after receiving 5 mSv of neutron radiation prior to the 50 mSv challenge dose. In contrast, it was less notable after receiving 10 mSv of neutron radiation prior to the 50 mSv challenge dose, which is consistent with the findings of Gajendiran et al.^11^. The adaptive response seen in the comet assay findings indicates the evidence of a resistance to the induction of DNA alterations following the irradiation of rats that were previously exposed to acute low doses of neutrons below the annual permissible doses with low dose rates.

The total Hb concentration after acute neutron doses showed a significant change, which differs from the insignificant change observed in gamma doses reported by Attia et al.^55^. This difference could be due to various factors such as differences in irradiation beam quality, type of radiation, and the range of dose investigated. Additionally, the change in the total Hb concentration was slight and further complicated by age differences in the studied animals. RDWs provided a more sensitive measure of small variations in red blood cell size than peripheral smears for detecting mild and moderate degrees of Iron Deficiency Anaemia (IDA). As such, RDWs could be used as a successful diagnostic technique for IDA RDWs can also express small variations in different populations of red cell size^56^ and correlates with the variation in red blood cell size (homogeneity or heterogeneity) equivalent to anisocytosis^57^. Thus, changes in the electrical properties of the cell membrane caused by oxidative damage by different free radicals could be responsible for the negative effects of radiation exposure on red blood cell shape and size distribution^58^. The study found increased values of MCV and RDWs in neutron exposure for 5, 10, and 50 mSv, indicating the presence of cells of widely differing sizes that might be a result of changes in the erythrocyte cell membrane. Oxygen-free radical species produced from exposure to ionizing radiation induce deleterious damage to the cell membrane. The group exposure to 5 mSv before exposure to dose (50 mSv) showed an adaptive response higher than the group exposure to 10 before 50 mSv irradiation as it reduced the membrane damage of 50 mSv exposure as shown in low MCV and RDWs results.

It is worth mentioning that RAR behavior can be noticed clearly in the G6 and G7 groups when compared with the RAR-control group (G5) in all studied parameters here except MCHC and HCT%. The enhancement of antioxidant parameters such as GSH, CAT, and SOD enzymes led to scavengers of free radicals, low production of lipid peroxidation, damage to DNA, and the volume and distribution width of red blood cells. This is an adaptation against thermal neutrons due to the exposure to priming doses of 5 and 10 mSv before the challenge dose of 50 mSv when compared to 50 mSv only (G5). The analysis of RAR factor values for all dosimetric markers under the study indicates that a lower priming dose leads to a higher adaptation response, while a higher priming dose results in the opposite effect. This suggests a dependence of the factor on the priming dose.

Ultimately, the fitting of the results reveals that the SOD, EPR, DNA%, RDWs, and Hb concentration parameters have a linear regression, while TL has a polynomial fitting. On the other hand, GSH, CAT, CA%, TM, OTM, and MCV responses to thermal neutrons can be exponentially fitted. All linearly fitted parameters with an R-value greater than $0.99$ are the best choice for the bio-dosimeters. However, they do not have the same precision; DNA% is the most precise biomarker, whereas EPR has the greatest standard deviation. Further studies are still required for a complete understanding of the adaptive response and the dosimetry for low doses of thermal neutrons, such as the duration of the intervals between irradiations, long-term stability, and storage temperature effect.

## Conclusion

Prolonged exposure to low levels of neutron radiation can result in elevated DNA damage, lipid peroxidation (MDA), free radicals, and mean corpuscle volume (MCV), as well as a decrease in antioxidants such as GSH, SOD, and CAT. The current research has identified DNA%, SOD enzymes, EPR intensity, total Hb concentration, and RDWs as biomarkers for assessing thermal acute neutron dose, with DNA% being the most effective biomarker for acute exposure. However, these suggested biomarkers need further investigations to confirm their reliability and applicability in the field of biodosimetry of thermal neutrons. The study also found that stimulation of the immune system occurred in the group exposed to 5 or 10 mSv prior to a 50 mSv challenge dose from thermal neutron, indicating that the rat was healed from the neutron radiation effect rapidly within two weeks. This has been proven by elevated concentrations of the antioxidants, a lack of free radicals, low lipid peroxidation, a reduction in the average volume of red blood cells (MCV), a narrowing of the red cell size distribution (RDWs), and a decrease in DNA damage, percentage of DNA in tail, and tail moment.

The radiation adaptive response behavior for the group (G6) exposed to 5 mSv prior to receiving a dose of 50 mSv is more obvious than it is for the group (G7) exposed to 10 mSv beforehand. It is worth noting that the lowest RARED, which was calculated as an acute neutron dose, is for catalase enzyme (CAT) at either 55 or 60 mSv, (5.6, 16.5 mSv) respectively, while the highest dose was for total Hb concentration (16.6, 40.9 mSv) respectively. The estimated average of RARED for both 55 and 60 mSv accumulated doses was about one-sixth and one-third of their actual doses, respectively. The calculated RAR factor indicates that the lower priming dose promotes a higher adaptation response and vice versa. The impact results of acute doses of thermal neutrons prompt us to investigate the effect of mixed fields of gamma and neutrons on biological systems. Despite this positive result of the radio-adaptive response, it is important to emphasize that acute doses of a neutron source still have harmful effects on some biological systems. Therefore, we strongly recommend carefully managing radioactive materials to prevent the harmful effects of ionizing radiation exposure, in line with the recommendations of international radiation organizations and committees.

## Data Availability

Data sets generated during the current study are available from the corresponding author on reasonable request.
